# A good gut feeling on Sarm1-mediated axon degeneration

**DOI:** 10.1007/s13238-021-00842-x

**Published:** 2021-04-26

**Authors:** Victoria E. von Saucken, David J. Simon

**Affiliations:** 1 Department of Biochemistry, Weill Cornell Medical College, 1300 York Avenue, New York, NY 10065 USA; 2 Tri-Institutional MD-PhD Program, Weill Cornell Medicine, Rockefeller University, Memorial Sloan Kettering Cancer Center, 1300 York Avenue, New York, NY 10065 USA

Axons, the cable-like projections of neurons, experience a wide range of stressors throughout their lifetimes. The result of any one of these challenges can be catastrophic, promoting degeneration of the axon. Axon degeneration is an active signaling process observed in traumatic brain injury, chemotherapy-induced peripheral neuropathies, and neurodegenerative disease (Simon and Watkins, 2018; Coleman and Hoeke, 2020). As a result, tremendous efforts have been made to identify biological targets that can be harnessed to block axon degeneration to treat these axonopathies. One promising candidate is Sarm1 (sterile alpha and TIR motif containing protein 1), a member of the Myd88 immune adaptor protein family that itself has no clear role in innate immunity. Sarm1 has emerged in fly and mouse as an essential signaling player in an axon-autonomous program of self-destruction (Osterloh et al., 2012; Loring and Thompson, 2019). *Sarm1* loss-of-function potently protects axons from injury-induced “Wallerian” degeneration and axon degeneration seen in models of chemotherapy-induced peripheral neuropathies (Geisler et al., 2016) and traumatic brain injury (Marion et al., 2019). While loss of Sarm1 is broadly protective, this protection is not universal, as evidenced by failure of the *Sarm1* knockout to protect against axon degeneration in a mouse model of amyotrophic lateral sclerosis (Peters et al., 2018). As our understanding of the pathophysiology of neurodegeneration improves, the question of why neurons contain activators of axon destruction remains unanswered. Could there be contexts in which axon degeneration is beneficial?

In this issue of *Protein* & *Cell*, Sun and Wang et al. discover a protective role of axon degeneration in inflammatory disease. Using a modified version of iDISCO, a method for whole tissue clearing and immunolabeling (Renier et al., 2014), the authors generate a 3D visualization of the axons composing the enteric nervous system within intact mouse, non-human primate, and human gut tissues (Sun et al., 2021). This technique allows Sun, Wang, and colleagues to visualize degeneration of catecholaminergic axons within the inflamed bowel in humans with ulcerative colitis and in mice treated with dextran sulfate sodium (DSS), a model of inflammatory bowel disease. To determine the functional effect of catecholaminergic axon degeneration on colitis, the authors evaluate DSS-treated *Sarm1* knockout mice and observe strong axon protection in the absence of Sarm1, consistent with catecholaminergic axon degeneration in colitis requiring Sarm1 activity.

Inhibition of axon degeneration in other disease models limits pathology, therefore it seemed likely that the physiological consequence of blocking the inflammatory death of enteric axons would lessen disease burden. Surprisingly, the authors discover the opposite; preservation of catecholaminergic axons following DSS treatment in *Sarm1* knockout mice enhances inflammation and crypt loss in the colon mucosa and increases body weight loss. The authors provide evidence that these effects are due to Sarm1 functioning in axons by generating a Sarm1 conditional knockout mouse to selectively delete the protein in catecholaminergic neurons. Here they observe axon protection and exacerbation of colitis similar to the full knockout. Since blocking axon degeneration worsens colitis, the authors hypothesize that impeding catecholaminergic axon function via chemogenetic inhibition or ablating these axons with 6-OHDA (a dopaminergic neuron toxin) would be beneficial in colitis. Indeed, both of these manipulations alleviate DSS-induced colitis. Contrary to prior studies showing that Sarm1-driven axon degeneration is pathological (Geisler et al., 2016; Marion et al., 2019), Sun and Wang et al. provide an exciting alternative view of Sarm1-driven axon degeneration protecting the colon against inflammation in a mouse model of inflammatory bowel disease.

The resolution of inflammation is protective in colitis, but how axon degeneration participates in ameliorating colitis is unresolved. This study reveals that the expression of key pro-inflammatory cytokines *IL-17A* and *IL-17F* is increased in DSS-insulted colon tissues in *Sarm1* knockouts, suggesting that the preservation of catecholaminergic axons promotes colon inflammation. To test this, the authors culture major IL-17 cytokine producers, T Helper 17 (Th17) cells and type 3 innate lymphoid cells (ILC3s), in the presence of the catecholaminergic neurotransmitter norepinephrine. In response to norepinephrine, these immune cells upregulate the transcription factor *RORγt* and its targets *IL-17A* and *IL-17F*. These data suggest that the preservation of catecholaminergic axons in *Sarm1* knockout mice enhances colon inflammation through sustained norepinephrine release, promoting IL-17A and IL-17F expression in nearby immune cells. It will be important to elucidate how norepinephrine, a well-known immunosuppressive agent (Sharma and Farrar, 2020), regulates these novel pro-inflammatory transcriptional changes in immune cells in future studies.

Given the clinical interest in modulating Sarm1 activity, a critical question is raised by this study: what causes Sarm1 activation and eventual axon degeneration in colitis? A recent study has provided a clue that the pro-inflammatory cytokine TNFα can lead to Sarm1 activation downstream of the activated necroptosis machinery in both retinal ganglion and sensory neurons (Ko et al., 2020). In agreement, Sun and Wang et al. show that TNFα treatment of cultured catecholaminergic neurons triggers axon degeneration that is suppressed by *Sarm1* deletion, and that blocking TNFα action on neurons via the administration of an anti-TNFα neutralizing antibody in mice protects axons from degeneration after DSS insult. Mechanistically, Sarm1 has been most studied in the setting of Wallerian degeneration, a process that involves axonal depletion of nicotinamide adenine dinucleotide (NAD^+^) and ATP (Wang et al., 2005; Gerdts et al., 2015; Yang et al., 2015). The authors show that catecholaminergic neurons behave similarly in response to TNFα with a local metabolic block preceding Sarm1-dependent axon degeneration. These data point to the local release of TNFα in the inflamed colon driving axonal Sarm1 activation and thereby triggering a well-described local energy deficit to drive axon destruction.

Altogether, Sun and Wang et al. prompt a reevaluation of axon degeneration as a harmful process by introducing a disease state in which axon degeneration is important to limit inflammation and disease burden in the gut. This highlights that the context of axon destruction is critical to understanding the cause and biological significance of neurodegeneration. Therefore clinical contexts may exist where therapeutic inhibition of this central pathway has unintended consequences, whether in the inflamed gut or in other organ dyshomeostases yet to be identified.
